# The association between study conditions and hair cortisol in medical students in Germany – a cross-sectional study

**DOI:** 10.1186/s12995-023-00373-7

**Published:** 2023-05-30

**Authors:** Meike Heming, Peter Angerer, Jennifer Apolinário-Hagen, Urs Markus Nater, Nadine Skoluda, Jeannette Weber

**Affiliations:** 1 Institute of Occupational, Social, and Environmental Medicine, Centre for Health and Society, Faculty of Medicine, Heinrich-Heine University Düsseldorf, Moorenstraße 5, 40225 Düsseldorf, Germany; 2 Department of Clinical Psychology and Health Psychology, Faculty of Psychology, University of Vienna, Liebiggasse 5, 1010 Vienna, Austria; 3 Research Platform The Stress of Life (SOLE) - Processes and Mechanisms underlying Everyday Life Stress, University of Vienna, Vienna, Austria

**Keywords:** HCC, Cortisol, Effort-reward imbalance, Job-demand-control support model, Medical students, Study conditions

## Abstract

**Background:**

Medical students often experience high levels of stress due to adverse study conditions, which may have adverse health consequences. Hair cortisol concentration (HCC) has been described as a physiological marker for chronic stress and might thus help to identify students under stress and examine the study conditions being responsible for long-term physiological stress responses. This study therefore investigated the association between study conditions and HCC in a sample of medical students.

**Methods:**

Fifty-five students from a medical school in Germany completed a paper-based questionnaire and had hair samples collected between July 2020 and July 2021. Study conditions were assessed with student versions of questionnaires based on the Job-Demand-Control-Support model (StrukStud, 25 items) and Effort-Reward Imbalance model (Student ERI, nine items). HCC of two centimeters closest to the scalp were determined by a cortisol luminescence immunoassay. Linear multiple regression analyses were performed to examine associations between study conditions and HCC.

**Results:**

Demands (B = 0.23, p = 0.002), effort (B = 0.12, p = 0.029) and the effort-reward-ratio (B = 0.28, p = 0.007) were positively associated with HCC in separate regression analyses, adjusted for age and sex. Only the association between demands and HCC remained significant when all components of the respective questionnaire were considered in the same model (B = 0.22, p = 0.003).

**Conclusion:**

The results suggest that adverse study conditions may be associated with activation of the hypothalamic-pituitary-adrenal axis stress response as reflected by increased HCC. Longitudinal research is needed to confirm these cross-sectional results and examine effects of more prolonged stress due to adverse study conditions.

**Supplementary Information:**

The online version contains supplementary material available at 10.1186/s12995-023-00373-7.

## Introduction

High stress levels have been observed among medical students [1, 2] and stressors related to the medical education rather than personal stressors were reported as the most important sources for stress [3]. The high stress level manifests itself from the beginning and can increase as studies progress or show peaks during the course of medical education [1, 4, 5]. In Germany, reported stress levels by medical students were found to be higher compared to the general population [2]. In addition, medical students also report high levels of depressive symptoms and anxiety [6–8]. To prevent potential health consequences of chronic stress during medical studies, it is therefore of utmost importance to identify adverse study conditions and persons at risk for stress-related diseases.

For some years now, the concentration of cortisol in hair has been discussed as a potential biological marker for chronic stress [9]. Within the physiological stress response the neuroendocrine pituitary-hypothalamic-adrenocortical (HPA) axis is activated and the glucocorticoid cortisol is secreted as an end hormone in the adrenal cortex [10]. Increased cortisol levels in response to acute stress are helpful in short term but can have negative health effects if they are repeatedly or constantly increased [10]. However, saliva or serum cortisol levels can only be measured at one time point and are varying throughout the day and therefore have rather been used to measure acute stress levels [9]. Instead hair cortisol concentration (HCC) reflects the cumulative secretion of cortisol for the respective period of hair length, and thus became a potential marker to measure chronic stress [9]. In contrast to self-report, biomarkers such as HCC might not only measure subjective stress levels, but also reflect stress-related activity of the HPA axis [11]. HCC might thus be used to early identify individuals at risk for stress-related diseases [9]. Although it is recommended to control for confounders such as age and sex, HCC seems to be robust to a set of other variables (i.e. medication use or smoking) which underlines the advantages and usefulness of HCC in research [12, 13]. Raul et al. (2004) were the first to develop a method to extract and determine HCC as a complementary method in doping controls [14]. HCC has further been investigated in relation to stress-related conditions [15], with increased HCC found in individuals suffering from chronic pain, in endurance athletes experiencing physical stress or in individuals having reported major life events, i.e. death of a relative, illness [16–18].

So far, research on HCC and its association with psychosocial stress is growing but entails mixed findings [11, 15, 19]. Among university students, HCC has been examined in relation to psychosocial stressors such as negative life events and in relation to perceived stress understood as a psychological response to stressors [16, 20–22]. While no associations were found between HCC and weekly assessed perceived stress and perceived stress during the past three months [16, 21, 23], some studies reported associations between HCC and serious or stressful life events during the past three months and weekly assessment [16, 22]. To the best of our knowledge, the relationship between adverse study conditions and HCC has not yet been examined. In regard of working populations, many studies have investigated two of the most prominent work stress models, the Job Demand Control Support (JDCS) and the Effort Reward Imbalance (ERI) model with HCC. In the JDCS model, it is assumed that adverse working conditions (e.g. high work demands, low control and low support from colleagues or superiors) can lead to work stress [24, 25]. The ERI model postulates that work stress can arise when employees put high efforts in their work but are not sufficiently rewarded for it [26]. While so far, no associations were found between the JDCS model and HCC [27–29], there were some studies that found positive correlations between HCC and the effort-reward ratio (ER-ratio) [30] and positive associations between effort, effort-reward ratio and HCC in a high workload sample [29]. Studies with longitudinal designs have shown that both positive and negative associations can be reported. For example, a study has shown that an increase of work stress in terms of the ERI model was associated with an increase in HCC in a small sample of 40 men working in lower and middle management positions [31]. A second prospective study has shown that a one-year increase in ER-ratio predicted a decrease in HCC at follow up two years later as well as a two-year decrease in terms of a change score in HCC [32]. Those contradicting findings might be explained by dysregulation of the HPA-axis after a prolonged exposure to chronic stress. Elevated cortisol levels are suggested to occur during an initial phase of chronic stress due to the activation of the HPA-axis [33]. However, after prolonged exposure to chronic stress, previous evidence suggests that the activity of the HPA-axis decreases, resulting in reduced cortisol levels [33].

Recently, questionnaires based on the two work stress models ERI and JDCS were adapted for students [34, 35]. Those questionnaires specifically measure study conditions (i.e. study demands, decision latitude, support from professors or lecturers and fellow students, efforts and rewards) that are assumed to be associated with study stress and adverse health [34–37]. Applying the ERI model to a university setting can be helpful in linking stress and health based on a theoretical model [38, 39]. Within university settings, a previous validation study thus defined effort as a great study load and rewards as being respected from professors or lecturers [38]. Subsequent studies confirmed the generalizability of the ERI model for university settings. For example, a qualitative study examining stressors and resources among medical students in Germany found that students perceive that the large amount of time spent studying for exams does not translate into appropriate grades [40]. Quantitative studies further found that an imbalance between efforts and rewards is associated with psychological distress and negative health outcomes [39, 41–43]. Also the JDCS model can be applied to the university context [35, 37]. Similar to the working context, demands for students were defined as having a high study load due to examinations or having time pressure [37]. Decision latitude for students can reflect strict study plans with fixed courses and without room for choosing examination topics [37]. Some studies confirmed the application of a JDCS model in students by showing that high demands and low decision latitude were associated with higher distress and by showing that higher demands were associated with lower satisfaction with academic or student life [44, 45].

To our knowledge, this study will be the first to investigate these validated questionnaires measuring study conditions in medical students and their association with HCC. It could therefore give first indications which factors in medical school might have stress-related consequences on a physiological level. This knowledge might help to identify risk groups among medical students, and study conditions that put them at risk. In the long run this will help to develop interventions on organizational and individual levels to prevent adverse health consequences such as cardiovascular diseases or depression. Due to the lack of knowledge on how study conditions may be associated with HCC, this study cannot formulate a directional hypothesis. Thus, the aim of this study is to explore whether study conditions are positively or negatively associated with HCC.

## Materials and methods

### Study design and participants

**Fig. 1:**
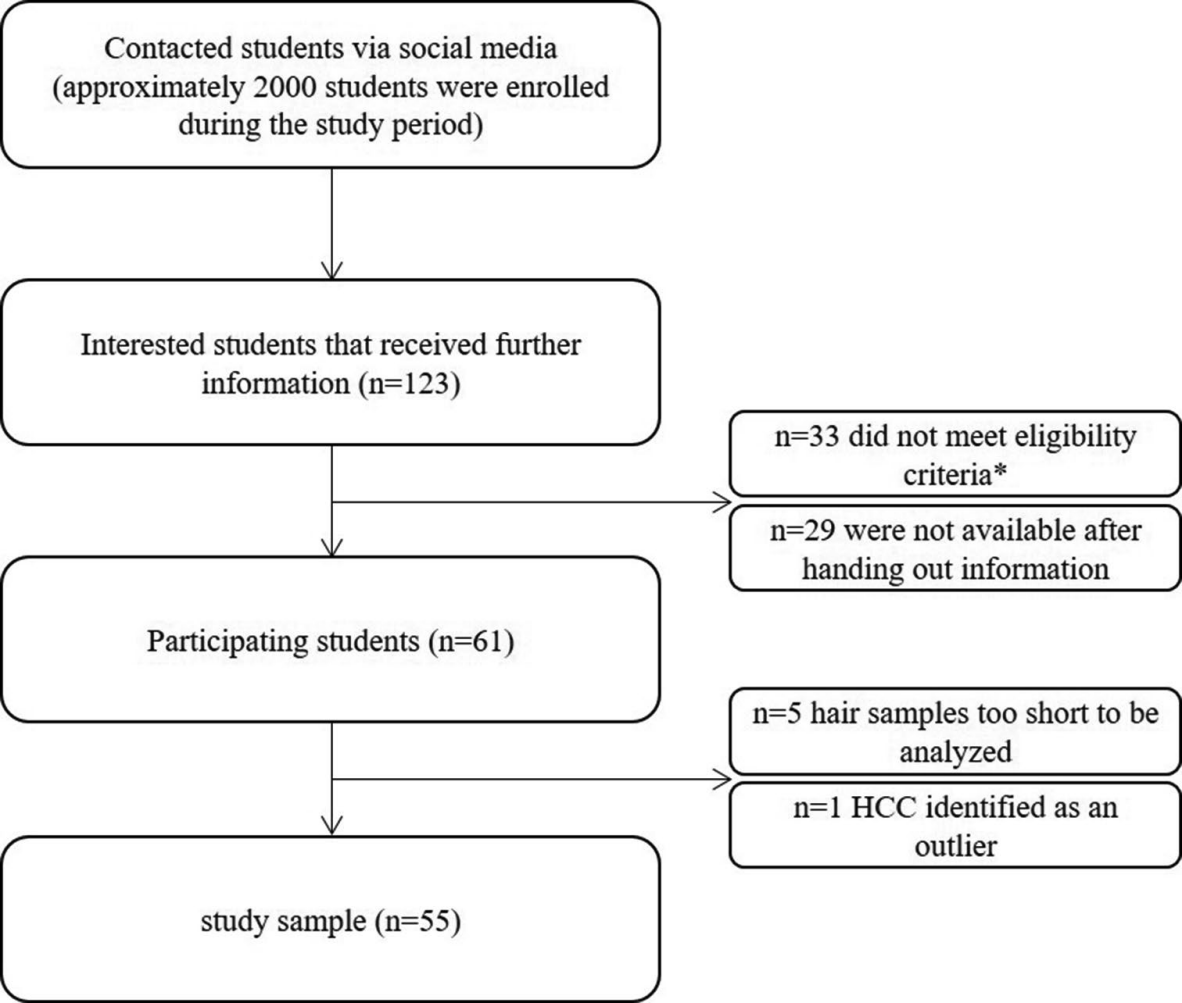
Flow chart of study sample. *Somatic or mental health conditions (n = 7), enrolment in the first or tenth semester or enrolment in human medical studies (n = 22), too short hair (n = 4)

This study used cross-sectional baseline data from a more elaborate ecological momentary assessment study called 3-S- (Stress, Strain, Stress reactivity) Student Study. This section only provides details of the present study (for detailed information about the entire study design see the study protocol https://osf.io/xkrz5). From July 2020 until July 2021, medical students (n = 61) who were enrolled at the Heinrich-Heine University Düsseldorf in Germany, participated in the study. The participants completed a baseline questionnaire, were exposed to a Trier Social Stress Test via Virtual Reality (VR-TSST [46]), had a hair sample taken, and participated in an ecological momentary assessment over three days. Participants were recruited using online flyers being distributed via social media (i.e. Facebook sites and WhatsApp groups of semester groups and the student body of the medical faculty of the Heinrich-Heine-University Düsseldorf). Interested students received further study and participant information upon request and were then able to decide whether they would like to participate in the study. Inclusion criteria were being enrolled in human medical studies of the Heinrich-Heine-University Düsseldorf between the second and ninth semester. Exclusion criteria were any health condition that might be associated with increased health risk during participation or that might contribute to cardiovascular stress reactivity (e.g., cardiovascular diseases, mental health disorders, hormonal disorders, lymphedema or other skin, bone or muscle disorders for which blood pressure measurements are contraindicated), heavy cigarette consumption (more than 10 per day), substance abuse and hair length of less than two centimeters. A detailed description of recruitment and participation in the study is shown in Fig. 1.

After enrollment, participants completed a paper-pencil questionnaire that assessed study conditions as well as sociodemographic factors, health status, and lifestyle questions. This took place in the facilities of the Institute of Occupational, Social and Environmental Medicine of the Heinrich-Heine-University Düsseldorf. To determine HCC a research assistant took hair strands from the participants with a length of at least two centimeters. Hair strands of five participants were too short (< 2 cm) for conducting analyses and were thus excluded from the sample (n = 56). One person was excluded after identifying the hair cortisol value as an outlier (exceeding three standard deviations from the mean, without any plausible biological reason) which restricted the final study sample to 55 participants. There were no missing data for other study variables.

Ethical approval for this study was obtained by the ethics committee of the medical faculty of the Heinrich-Heine University of Düsseldorf (2019 − 714). All participants gave written informed consent to participate in the study.

### Measures

#### Job demand control support model (JDCS) in university setting

Based on the well-established JDCS model [24] and the corresponding Job-Content questionnaire [25], Schmidt et al. (2019) developed a questionnaire (in German: StrukStud) that measures study conditions [35]. The questionnaire had been validated in four study samples and showed good psychometric properties [35]. It includes four scales formed by a total of 25 items and was answered by participants on a 4-point Likert scale from 1 (not applicable) to 4 (applicable). Demands were measured by seven items and had acceptable internal consistency (Cronbach’s alpha 0.71). Decision latitude entailed five items for skill discretion and three items for decision authority and had sufficient internal consistency (Cronbach’s alpha 0.71). Social support was measured with one scale for support from lecturers/professors (five items, Cronbach’s alpha 0.77) and one scale for support from fellow students (five items, Cronbach’s alpha 0.77). Both scales had acceptable internal consistency. Average scores were calculated. Higher scores indicate higher demands, a high decision latitude and more social support from fellow students or lecturers/professors. An item example for demands is “In my studies I have to work fast” [35]. One example for a decision authority item is “My studies include the opportunity to have a say” and an example item for a social support item is “My fellow students help me in my studies” [35]. The translated version of all items is given in the additional material (Additional file 1) [35].

#### Effort reward imbalance (ERI) in university setting

In addition, a previous developed and validated Student-ERI questionnaire was applied in this study [34]. Participants answered nine items on a 4-point Likert scale from 1 indicating strong disagreement to 4 indicating strong agreement. Effort was measured by three items and had poor internal consistency (Cronbach’s alpha 0.50). An example of an effort item is “I have constant time pressure due to a heavy study load” [34]. Reward was measured by six items and had questionable internal consistency (Cronbach’s alpha 0.67). An item example of a reward item is “Considering all my efforts, I receive the appreciation that I deserve” [34]. The translated version of all items, originally published by Wege et al. (2017), is given in the additional material (Additional file 2) [34]. Higher scores represent higher effort and more rewards. Average scores were calculated for the two scales and the effort score was divided by the reward score to calculate the effort-reward ratio. A value below one indicates a favorable condition as there is less effort for rewards. A value equal to one indicates a balance between effort and reward and a value beyond one indicates an imbalance, as more efforts for rewards are reported [47].

#### Hair cortisol analysis

The hair samples of the study participants were taken close to the scalp from the posterior vertex region of the head at the same day they completed the baseline questionnaire. Previous studies have acknowledged interindividual variance in hair growth (e.g. due to ethnicity or sex), ranging from 0.6 to 1.4 cm per month [48] or 0.7 to 3.6 cm per month [49]. Since the assessment of individual hair growth is less feasible in the field, researchers agreed on average hair growth of one cm per month for hair testing [50]. Therefore, the first two centimeters closest to the scalp were used to determine HCC which reflect the cumulative cortisol secretion during the last two months [49]. Hair analysis was conducted in Vienna, Austria, following laboratory protocol described by Goreis et al. (2022), with using 10 mg finely cut hair  (for further details, see [51]). Cortisol levels were determined by using a commercially available cortisol luminescence immunoassay (LIA; IBL International, a Tecan Group company, Hamburg, Germany). Inter- and intra-assay coefficients of variation were below 10%.

### Statistical analyses

To achieve a statistical power of 0.8, a p-level of 0.05 with a medium effect size (f^2^) for one predictor, 54 participants are sufficient [52]. A medium effect size was previously estimated by Staufenbiel et al. (2013) who calculated and showed medium to large effect sizes for chronic stressors on HCC in their review [15].

HCC were not normally distributed, assessed by the Shapiro-Wilk-Test, p < 0.05. Therefore HCC data were log_10_-transformed to approach a normal distribution. Log_10_-transformed HCC were approximately normally distributed, assessed by Shapiro-Wilk-Test, p > 0.05.

To examine whether adverse study conditions are reflected by increased HCC, separate multiple linear regressions were performed. Separate regression analyses were used to identify if any of the applied scales were associated independently with HCC.

First, study conditions, i.e. demands, decision latitude, support from professors and support from students were set each as the independent variable and HCC was set as the dependent variable.

Second, study conditions in terms of effort, reward and ER-ratio were set each as the independent variable and HCC as the dependent. In a second step, potential confounders including age (continuously) and sex (male/female) were added to each model [13, 19]. As only one person reported smoking, and sensitivity analyses without this person did not show different results, smoking was not excluded. Medical students in Düsseldorf take an intermediate medical examination at the end of the third study year, which yields the possibility that students in the third year (or 6th term) might experience higher stress levels. However, study year did not correlate with HCC and was therefore not included as a confounder. Previous studies on psychology students reported as well that study year does not explain study-related stress [37, 44]. An additional table on the mean values of study conditions and HCC by study year can be found in the additional material (Additional file 3). Some other potential confounders (i.e. body mass index, contraceptive use in women (yes/no), alcohol consumption (never; once a month; 2–4 times a month; 2–3 times a week; 4 times a week) or physical activity (less than 1 h a week; 1–2 h a week; 3–4 h a week; 5–6 h a week; more than 6 h a week) did not correlate with HCC in our study sample and were therefore not considered as confounders in this study. It is also suggested that some specific factors related to hair may be influencing HCC [12]. So far, factors such as hair dye, hair color or frequency of hair washing were found to be influencing HCC only in animals, but not in humans [12, 14, 53]. Frequency of hair washing (times per week) or dyed hair (yes/no) did also not correlate with HCC in our study sample and were therefore not adjusted for in the analyses.

Additionally, multiple linear regression analyses were conducted, where all components of the JDSC were put into the same model with HCC as the dependent variable. This was done accordingly for the components of the ERI model. These additional analyses were performed to determine whether associations with HCC persisted and which items contributed the most to the association when the full theoretical construct of the respective model was considered. Since the two models are based on different theoretical frameworks but show moderate and high correlations with each other in some cases, we decided to not analyze them in a joint model but to consider them individually. The analyses were adjusted for age and sex.

Results are presented as B-values (unstandardized regression coefficients) with 95% confidence intervals (CIs). Statistical significance was assumed at a p-level below 0.05. Pearson correlation analyses of the independent and dependent study variables can be found in Additional file 4. Furthermore, we divided our independent variables according to their median into high and low exposure groups and performed two-tailed t-tests for HCC mean levels. With this we give additional information on potential statistical differences in HCC mean values for the independent variables. Post-hoc statistical power calculations for multiple regression based on p-level of 0.05, observed R^2^, sample size and number of predictors were performed online with a post-hoc statistical power calculator for multiple regression [54]. Among other things, this can help to carefully interpret non significant results [55]. All other analyses were performed with IBM SPSS Statistics, version 25.

## Results

### Descriptive statistics

Characteristics of the study sample are presented in Table 1. Among the study participants were 42 females (76.4%) and 13 males (23.6%). Mean age of the medical students was 22 years (range from 19 to 31 years). They were enrolled between the second and ninth semester with most of the students being enrolled in a higher semester and only nine being enrolled in the first or second study year (16.4%). The students had HCC mean of 5.58 pg/mg. Mean levels of HCC were similar for men and women (M = 5.3, SD = 3.51 and M = 5.67, SD = 2.81, p = 0.979, data not shown).

It was also observed, that students who were enrolled in their third study year reported somewhat higher demand or ER-ratio mean values than students enrolled in the fourth or fifth study year (Additional file 3). HCC mean levels were the highest in the first and second study years (Additional file 3).

**Table 1:** Descriptive statistics of the study sample (n = 55)

	n (%)	Mean	SD^a^
**Sex**			
Female	42 (76.4)		
Male	13 (23.6)		
**Age**			
19–31		22.18	2.11
**Study year**		3.65	1.21
1 (Semester 2)	3 (5.5)		
2 (Semester 3–4)	6 (10.9)		
3 (Semester 5–6)	16 (29.1)		
4 (Semester 7–8)	12 (21.8)		
5 (Semester 9)	18 (32.7)		
**JDCS in university setting** ^**b**^ scale range 1–4			
Demands		3.09	0.44
Decision latitude		2.83	0.40
Support from students		3.69	0.37
Support from professors/lecturers		2.47	0.54
**Student ERI** ^**c**^ scale range 1–4			
Effort		2.6	0.61
Reward		3.12	0.45
Effort-Reward-Ratio (effort/reward)		0.87	0.31
**HCC** ^**d**^ pg/mg			
1.60-15.43		5.58	2.96
**HCC** ^**d**^ log-transformed			
0.20–1.19		0.70	0.24

^a^Standard deviation

^b^Structural study conditions questionnaire (In German: StrukStud)

^c^Student version of effort-reward imbalance questionnaire

^d^Hair cortisol concentration

### Regression analyses

Table 2 shows the results of separate linear regression analyses for study conditions in medical students and logarithmic transformed HCC. All analyses were adjusted for sex and age.

### JDCS in university setting and HCC

There was a significant positive association between demands and HCC (B = 0.23, p = 0.002, Model 1^b^, Table 2), meaning that medical students who reported higher demands in their studies also had higher HCC compared to medical students with lower demands. Students who reported to have poorer social support from their fellow students had higher HCC compared to students who reported to have better social support from their fellow students. However, this association was weak and did not reach significance. Other single components of the JDCS model, namely decision latitude and support from professors/lecturers, were not associated with HCC.

Analyzing all components of the JDCS model and HCC in the same model showed that demands remained to be significantly associated with HCC (B = 0.22, p = 0.003, Table 3). Within this model, no other components of the JDCS model in university setting were significantly associated with HCC.

### Student ERI and HCC

Effort was significantly associated with HCC (B = 0.12, p = 0.029, Model 1^b^, Table 2). Medical students who reported higher efforts also had higher HCC than students who reported lower efforts in their studies. While there was no association between reward and HCC, there was an association found between ER-ratio and HCC. Students who had a greater imbalance of effort and reward in their studies had higher HCC compared to students with a lower imbalance of efforts and rewards (B = 0.28, p = 0.007, Model 1^b^).

In the analysis examining both effort and reward within one model, the regression coefficient of effort decreased marginally and the association no longer reached significance (B = 0.11, p = 0.057, Table 3). Within this model, reward was not associated with HCC.

**Table 2:** Results from separate multiple linear regression analyses estimating the association between study conditions and HCC (n = 55)

	HCC^a^							
	Crude Model				Model 1^b^			
	B^c^	p-value	95% CI	SP^d^	B^c^	p-value	95% CI	SP^d^
**JDCS in university setting** ^**e**^								
Demands	**0.22**	0.002	0.09;0.35	0.916	**0.23**	0.002	0.09;0.36	0.826
Decision latitude	-0.02	0.815	-0.18;0.14	0.046	-0.01	0.860	-0.18;0.14	0.082
Support from students	-0.14	0.096	-0.31;0.03	0.407	-0.14	0.117	-0.31;0.04	0.286
Support from professors/lecturers	-0.02	0.796	-0.13;0.1	0.046	-0.02	0.739	-0.14;0.1	0.089
**Student ERI** ^**f**^								
Effort	**0.12**	0.02	0.02;0.22	0.681	**0.12**	0.029	0.01;0.22	0.5
Reward	-0.09	0.175	-0.23;0.04	0.279	-0.09	0.19	-0.23;0.05	0.219
Effort-Reward-Ratio	**0.28**	0.005	0.09;0.47	0.844	**0.28**	0.007	0.08;0.47	0.694

^a^Log-transformed hair cortisol concentration

^b^Adjusted for age and sex

^c^Unstandardized regression coefficients

^d^Statistical Power

^e^Structural study conditions questionnaire

^f^Student version of effort-reward imbalance questionnaire. Bold values indicate a p-value below 0.05

**Table 3:** Results from multiple linear regression analyses estimating the association between study conditions and HCC (n = 55)

	HCC^a^			
	Model 2^b^			
	B^c^	p-value	95% CI	SP^d^
**JDCS in university setting** ^**e**^				0.8
Demands	**0.22**	0.003	0.08;0.37	
Decision latitude	0.05	0.586	-0.12;0.22	
Support from students	-0.11	0.179	-0.28;0.05	
Support from professors/lecturers	-0.01	0.875	-0.04;0.02	
**Student ERI** ^**f**^				0.495
Effort	0.11	0.057	0.00;0.21	
Reward	-0.06	0.434	-0.20;0.09	

^a^Log-transformed hair cortisol concentration

^b^Adjusted for age and sex

^c^Unstandardized regression coefficients

^d^Statistical Power

^e^Structural study conditions questionnaire (In German: StrukStud)

^f^Student version of effort-reward imbalance questionnnaire. Bold values indicate a p-value below 0.05

### Additional analyses

The post-hoc calculated statistical power ranged from 0.046 to 0.916 in the crude models and from 0.082 to 0.826 in the adjusted analyses (Tables 2 and 3). Three models reached a statistical power above 0.8: The unadjusted Crude Model investigating the association between demands and HCC, the adjusted model of the same association and the unadjusted Crude Model investigating the association between the ER-ratio and HCC.

Additional results of two-tailed t-tests for HCC mean values by the JDCS in university setting and Student ERI variables are shown in Additional file 5. There was a significant difference in HCC mean scores for high demands (M = 0.74, SD = 0.21) and low demands (M = 0.56, SD = 0.23, t(53) = 2.83, p = 0.006). There were also significant differences in HCC mean values for high support and low support, and high efforts and low efforts. HCC mean values were higher in the group of low support and high efforts. There was an almost significant difference in HCC mean values for high ER-ratio (M = 0.75, SD = 0.24) and low ER-ratio (M = 0.32, SD = 0.20, t(53) = 2.00, p = 0.051). No other significant differences were observed.

## Discussion

This study aimed to investigate study conditions in medical school and their association with HCC as a biological marker for chronic stress. It was found that demands, efforts and an imbalance between efforts and rewards were positively associated with HCC in separate regression analyses. In regression analyses containing all components of the respective model, only the association between demands and HCC remained significant.

Contrary to our findings, three cross-sectional workplace studies failed to show associations between HCC and components of the JDCS model [27–29]. Those different findings might be explained by the varying study populations. For example, it was argued either the small number of workers with high demands [27] or the fact that demands at work might be perceived as stressful only for a short time period [28] might explain null findings among these workplace studies. In contrast, it was shown that high and constant study workload is one of the greatest stressors among medical students [1, 56]. As it is suggested that a certain level of stress must be reached for significant HCC changes to occur [19], this result suggests that medical students might perceive demands during their studies as so stressful and enduring that this is reflected in increased HCC. Other single components of the JDCS questionnaire including decision latitude and social support from professors or lecturers were not associated with HCC in the present study. On average, students reported to have less support from their professors or lecturers than from their fellow students. We did, however, observe a negative association between support from fellow students and HCC that almost reached significance. Considering the low statistical power and small sample size, this association might thus be of practical relevance and could indicate that support from fellow students is more relevant for students than support from professors or lecturers for physiological stress levels. This is in accordance to an earlier study giving evidence that support from fellow students could minimize psychological distress [57]. The lack of direct association between decision latitude and HCC is in accordance with findings of two preliminary studies, which also used the same questionnaire as the study at hand and failed to find a direct association between decision latitude and self-reported stress [37, 44]. Thus, the results suggest that low levels of decision latitude during medical studies might not be associated with chronic stress levels, physiologically reflected by HCC. Other workplace studies also failed to find associations between decision latitude and HCC [28, 29]. However, there might also be another explanation for this null finding: An experimental study suggested that some individuals can perceive increased autonomy negatively resulting in increased physiological stress responses [58]. Increased autonomy may come with a greater burden for some individuals, as additional decisions must be made [58]. These negative perceptions of increased autonomy could compensate responses from individuals for whom increased decision latitude acts as a resource to cope with high study demands. An additional approach to examine the association between JDCS in university setting and HCC could have been to calculate interactions between demands and decision latitude. This could reflect the JDCS model where not only high and low strain but also active conditions in terms of high decision latitude and high demands or passive conditions in terms of low decision latitude and low demands are reflected [25]. With this it could be investigated if certain exposures of decision latitude can change the extent of an association between demands and HCC. Unfortunately, our statistical power was not sufficient to calculate interaction analyses. However, to focus only on interaction results is also suggested to be too narrow and practical implications are similar for main effects and interactive effects [25, 59].

This study also found significant positive associations between effort and HCC and ER-ratio and HCC in separate linear regression analyses. The results are in line with previous cross-sectional findings among working populations [29, 30]. Thus, rewards including appreciation of academic performance by lecturers, fellow students and parents and appropriate performance appraisal might act as a resource helping medical students to balance high efforts. In turn, low levels of rewards might even enhance physiological stress responses of high efforts.

This study could show that adverse study conditions are associated with physiological stress levels among medical students with demands showing the strongest association with HCC. Furthermore, there was support for the theoretical concept of the ERI model, which states that an imbalance of efforts and rewards can lead to stress [26]. Those results extend previous research among working populations tending to support associations between efforts, the ER-ratio and HCC but not between demands and HCC (e.g. [11]).

Since we have analyzed two cm of hair strands closest to the scalp, the HCC of this study reflects cortisol secretion during the past two months [49]. One might thus hypothesize, that adverse study conditions trigger chronic or recurrent activation of the HPA stress response. Chronic activation of stress response systems might eventually lead to stress-related diseases due to allostatic load, also known as the wear and tear of the body due to accommodation to chronic stress [60]. Adverse study conditions might thus contribute to an increased risk of cardiovascular diseases in the long run and mental and other stress-related diseases among medical students in a shorter term. However, longitudinal studies are needed to confirm a causal relationship.

### Limitations

There are limitations that need to be addressed within this study.

First, as our study sample was relatively small there is a possibility that small effects, such as for social support, were not detected. However, statistical power of the analyses between demands and HCC were for example sufficient.

Second, only medical students from one big medical school were included which limits the possibility to generalize our findings to medical students of other medical schools and countries. However, similar results may be found in other countries or medical schools, as medical students report high stress levels also in other countries [1]. It is further suggested to adjust for ethnicity when analyzing HCC [49], but unfortunately we did not assess ethnicity of the participants in the current study.

Due to our sampling method, there is a potential that especially students interested in stress, responded to our request to participate in the study. For example, results would be overestimated, if only students participated that felt especially stressed. It may also be, that these particular students did not participate due to a lack of time to participate, which would underestimate our results. However, as only a small amount of students needed to be reached for the entire study design other sampling methods were not considered suitable.

Third, the measurement time points of the questionnaires and the hair sampling were not aligned. While hair samples reflected the past two months before data collection, the JDCS questionnaire did not provide definitions or introductory sentences on time frames. In addition, the ERI questionnaire captured the current study situation. Those operationalizations decreased the risk of recall bias, but we cannot exclude the possibility that study conditions have changed over the last two months. Furthermore, measurements that capture duration of adverse study conditions might be relevant as cortisol secretion might change with enduring chronic stress [33]. Also, the cross-sectional study design does not allow to detect temporal associations between study conditions and HCC. This is an important limitation of this study, as we cannot make causal statements whether study conditions predict HCC or if HCC, or rather third variables that increased HCC, also changed the perception of study conditions.

Fourth, the student ERI used in this study was validated by previous studies [34, 38], but the effort scale had poor internal consistency in the study at hand. This might have occurred due to the second effort item which showed poor corrected item-total correlation (< 0.3, “I have many interruptions and disturbances while preparing for my exams” [34]). Furthermore, the effort scale only includes three items, but internal reliability usually increases with the number of items added [61]. An adapted Italian version noticed similar problems with the second item and excluded it from analyses [38]. As other studies supported the original version of the student ERI [39, 62] and as a scale of only two items did not result in far better values for internal consistency, it was decided to keep the items as suggested by Wege et al. [34].

### Implications

By showing that adverse study conditions are positively associated with HCC among medical students, this study suggests that these associations should be further investigated by prospective studies. While some studies have shown that the increase in ER-ratio could lead to decreased HCC [32], others claim that it leads to increased HCC or no association at all [31]. Therefore, it still remains unclear, when and to what extent cortisol concentration changes if stress is perceived permanently [33, 63]. In our healthy study sample (i.e. without any diagnosed mental health disorders), we observed higher mean HCC in the first, second and third study year, lower mean HCC in the fourth study year and again higher mean HCC in the fifth study year. One study has found lower HCC in individuals diagnosed with general anxiety disorder compared to healthy controls [64]. With these descriptive and cross-sectional findings we are thus not able to draw conclusions on the two stage process of cortisol, but would like to emphasize that future prospective studies with a repeated measures design and bigger sample size are needed to investigate how prolonged adverse study conditions could alter changes in cortisol levels.

As we have shown that the applied theoretical work stress models for medical students are associated with physiological stress levels, further studies are warranted on adverse study conditions to find out more about the origin of stress in students [37]. It is further suggested, that interventions should focus on the organizational instead of individual level in order to reduce psychosocial stress in students [65]. Flexible rules for absence (and reduction of attendance times [66]) or more flexible scheduling are examples of such setting-based interventions [65]. Our findings also give indications for starting points to reduce students stress level by taking into account the study conditions as formulated in the work stress models JDCS and ERI. For example, students reported unfair grading systems that led to perceived imbalances of effort put into studying for exams and reward in terms of grades [40]. Next to this, additional courses early in the curriculum seem to help to compensate high study demands. For example, one study could show that time management trainings for students seemed to result in perceiving external demands (i.e., high workload) as less harmful compared to students who had no management training [67].

## Conclusions

This study expanded the knowledge about associations between study conditions and HCC in a sample of medical students in Germany. Effort, demands and imbalance between efforts and rewards were positively associated with HCC. Our findings indicate which study conditions are positively associated with physiological stress levels. Our findings warrant longitudinal research and indicate the need for taking a closer look at potential health effects of study conditions. Furthermore, this study strengthens the idea that HCC can be used as a marker to detect physiological stress due to adverse study conditions.

## Electronic supplementary material

Below is the link to the electronic supplementary material.

Additional file 1. Translated version of the StrukStud Items (translation provided by the authors of Schmidt et al. 2019).

Additional file 2. Translated version of the Student ERI Items, originally published by Wege et al. 2017.

Additional file 3. Descriptive statistics of the study variables by study year (n=55).

Additional file 4. Pearson correlations of independent and dependent study variables (n=55).

Additional file 5. Differences in mean values of HCC_log_: results from two-tailed t-tests (n=55).

## Data Availability

The datasets generated and/or analysed during the current study are not publicly available due to national data protection regulations.
