# Supplementary material for: The association between study conditions and hair cortisol in medical students in Germany – a cross-sectional study
Source: J Occup Med Toxicol. 2023 May 30;18:7. doi: 10.1186/s12995-023-00373-7 (PMC10228133; doi:10.1186/s12995-023-00373-7)
Supplement: Supplementary file 1 — Additional file 1. Translated version of the StrukStud Items (translation provided by the authors of Schmidt et al. 2019). [file 12995_2023_373_MOESM1_ESM.pdf]

The association between study conditions and hair cortisol in medical students in Germany – a cross-sectional study

Journal of Occupational Medicine and Toxicology

Meike Heming, Peter Angerer, Jennifer Apolinário-Hagen, Urs Markus Nater, Nadine Skoluda, Jeannette Weber

Corresponding author: Jeannette Weber, Institute of Occupational, Social, and Environmental Medicine, Centre for Health and Society, Faculty of Medicine, Heinrich-Heine University Düsseldorf, Universitätsstr. 1, 40225 Düsseldorf, Germany

Additional file 1. Translated version of the StrukStud Items (translation provided by the authors of Schmidt et al. 2019 (1)).

	In my studies…
Dem1	… I have to work fast.
Dem2	… I have to work hard.
Dem3	… I have to work excessively.
Dem4	… I have enough time for my tasks.
Dem5	… tasks compete with each other.
Dem6	… high concentration is required.
Dem7	… there is a hectic pace.
Skilldis1	… I learn many new things.
Skilldis2	… creativity is required.
Skilldis3	… I need a high skill level.
Skilldis4	… I have variety.
Skilldis5	… I develop my own skills.
	My studies…
Decaut1	… allow me to make my own decisions.
Decaut2	… offer little decision freedom. *
Decaut3	… include the opportunity to have a say.
	My lecturers/my professors…
Suppprof1	… care/take interest.
Suppprof2	… pay attention.
Suppprof3	… are repellent.*
Suppprof4	… help me in my studies.
Suppprof5	… support through good organization.
	My fellow students…
Supstu1	… are interested in me.
Supstu2	… are repellent.*
Supstu3	… are friendly.
Supstu4	… and I work together well.
Supstu5	… help me in my studies.

* Reversed Items.

References

1. Schmidt LI, Scheiter F, Neubauer A, Sieverding M. [Demands, Decision Latitude, and Stress Among University Students: Findings on Reliability and Validity of a Questionnaire on Structural Conditions (StrukStud) Based on the Job Content Questionnaire]. Diagnostica 2019; 65(2):63–74.
