# Supplementary material for: The association between study conditions and hair cortisol in medical students in Germany – a cross-sectional study
Source: J Occup Med Toxicol. 2023 May 30;18:7. doi: 10.1186/s12995-023-00373-7 (PMC10228133; doi:10.1186/s12995-023-00373-7)
Supplement: Supplementary file 2 — Additional file 2. Translated version of the Student ERI Items, originally published by Wege et al. 2017. [file 12995_2023_373_MOESM2_ESM.pdf]

The association between study conditions and hair cortisol in medical students in Germany – a cross-sectional study

Journal of Occupational Medicine and Toxicology

Meike Heming, Peter Angerer, Jennifer Apolinário-Hagen, Urs Markus Nater, Nadine Skoluda, Jeannette Weber

Corresponding author: Jeannette Weber, Institute of Occupational, Social, and Environmental Medicine, Centre for Health and Society, Faculty of Medicine, Heinrich-Heine University Düsseldorf, Universitätsstr. 1, 40225 Düsseldorf, Germany

Additional file 2. Translated version of the Student ERI Items, originally published by Wege et al. 2017 (1).

I have constant time pressure due to a heavy study load (eff1).
I have many interruptions and disturbances while preparing for my exams (eff2).
My study load has become more and more demanding (eff3).
I receive the respect I deserve from my supervisors (teachers) (rew1).
I receive the respect I deserve from my fellow students (rew2).
I am treated unfairly at university (rew3).*
I am not sure whether I can successfully accomplish my university training s (rew4).*
Considering all my efforts, I receive the appreciation that I deserve (rew5).
Considering all my efforts and achievements, my job promotion prospects are adequate (rew6).

* Reversed Items.

Reprinted from Wege N, Li J, Muth T, Angerer P, Siegrist J, Student ERI: Psychometric properties of a new brief measure of effort-reward imbalance among university students, 94:64-67, Copyright (2017) with permission from Elsevier.

References

1. Wege N, Li J, Muth T, Angerer P, Siegrist J. Student ERI: Psychometric properties of a new brief measure of effort-reward imbalance among university students. Journal of psychosomatic research 2017; 94:64–7.
