# Supplementary material for: The association between study conditions and hair cortisol in medical students in Germany – a cross-sectional study
Source: J Occup Med Toxicol. 2023 May 30;18:7. doi: 10.1186/s12995-023-00373-7 (PMC10228133; doi:10.1186/s12995-023-00373-7)
Supplement: Supplementary file 3 — Additional file 3. Descriptive statistics of the study variables by study year (n=55). [file 12995_2023_373_MOESM3_ESM.pdf]

The association between study conditions and hair cortisol in medical students in Germany – a cross-sectional study

Journal of Occupational Medicine and Toxicology

Meike Heming, Peter Angerer, Jennifer Apolinário-Hagen, Urs Markus Nater, Nadine Skoluda, Jeannette Weber[1]

Corresponding author: Jeannette Weber, Institute of Occupational, Social, and Environmental Medicine, Centre for Health and Society, Faculty of Medicine, Heinrich-Heine University Düsseldorf, Universitätsstr. 1, 40225 Düsseldorf, Germany

Additional file 3. Descriptive statistics of the study variables by study year (n=55).

	**Study year**									
	**1 (n=3)**		**2 (n=6)**		**3 (n=16)**		**4 (n=12)**		**5 (n=18)**	
	Mean	SD[a]	Mean	SD[a]	Mean	SD[a]	Mean	SD[a]	Mean	SD[a]
**JDCS in university setting[b]** scale range 1-4										
Demands	3.38	.54	3.26	.66	3.40	.39	3.19	.46	2.87	.31
Decision latitude	3.13	.33	3.00	.32	2.73	.28	2.78	.46	2.83	.48
Support from students	3.67	.31	3.53	.45	3.83	.25	3.78	.32	3.58	.43
Support from professors/ lecturers	2.47	.23	2.77	.32	2.50	.51	2.52	.54	2.31	.64
**Student ERI[c]** scale range 1-4										
Effort	2.78	1.07	2.83	.69	2.94	.30	2.31	.59	2.39	.60
Reward	3.22	.67	3.08	.48	3.08	.49	3.01	.45	3.20	.41
Effort-Reward-Ratio (effort/reward)	.94	.57	.94	.27	1.00	.34	.78	.22	.77	.25
**HCC[d]** pg/mg	6.5	1.20	6.25	2.93	5.96	3.61	4.79	1.91	5.38	3.20
**HCC[d]** log-transformed	.81	.08	.75	.25	.71	.24	.65	.19	.65	.27

[a] Standard deviation.

[b] Structural study conditions questionnaire (In German: StrukStud) (1).

[c] Student version of effort-reward imbalance questionnaire (2).

[d] Hair cortisol concentration.

References

1. Schmidt LI, Scheiter F, Neubauer A, Sieverding M. [Demands, Decision Latitude, and Stress Among University Students: Findings on Reliability and Validity of a Questionnaire on Structural Conditions (StrukStud) Based on the Job Content Questionnaire]. Diagnostica 2019; 65(2):63–74.

2. Wege N, Li J, Muth T, Angerer P, Siegrist J. Student ERI: Psychometric properties of a new brief measure of effort-reward imbalance among university students. Journal of psychosomatic research 2017; 94:64–7.
