# Supplementary material for: The association between study conditions and hair cortisol in medical students in Germany – a cross-sectional study
Source: J Occup Med Toxicol. 2023 May 30;18:7. doi: 10.1186/s12995-023-00373-7 (PMC10228133; doi:10.1186/s12995-023-00373-7)
Supplement: Supplementary file 4 — Additional file 4. Pearson correlations of independent and dependent study variables (n=55). [file 12995_2023_373_MOESM4_ESM.pdf]

**The association between study conditions and hair cortisol in medical students in Germany – a cross-sectional study**

**Journal of Occupational Medicine and Toxicology**

**Meike Heming, Peter Angerer, Jennifer Apolinário-Hagen, Urs Markus Nater, Nadine Skoluda, Jeannette Weber**

Corresponding author: Jeannette Weber, Institute of Occupational, Social, and Environmental Medicine, Centre for Health and Society, Faculty of Medicine, Heinrich-Heine University Düsseldorf, Universitätsstr. 1, 40225 Düsseldorf, Germany

**Additional file 4**. Pearson correlations of independent and dependent study variables (n=55).

| | Demands | Decision latitude | Support students | Support professors | Effort | Reward | ER- ratio[a] | HCC[b] |
|---|---|---|---|---|---|---|---|---|
| Demands | 1 | | | | | | | |
| Decision latitude | -.222 | 1 | | | | | | |
| Support students | -.109 | .092 | 1 | | | | | |
| Support professors | -.028 | .422** | .12 | 1 | | | | |
| Effort | .450** | -.270* | -.038 | -.072 | 1 | | | |
| Reward | -.337* | .465** | .29* | .578** | -.269* | 1 | | |
| ER-ratio[a] | .514** | -.385** | -.191 | -.353** | .805** | -.713** | 1 | |
| HCC[b] | .414** | -.032 | -.227 | -.036 | .312* | -.186 | .374** | 1 |

[a] Effort-Reward-Ratio.

[b] Hair cortisol concentration.

* $p < 0.05$ (two-tailed).

** $p < 0.01$ (two-tailed).
