# Supplementary material for: The association between study conditions and hair cortisol in medical students in Germany – a cross-sectional study
Source: J Occup Med Toxicol. 2023 May 30;18:7. doi: 10.1186/s12995-023-00373-7 (PMC10228133; doi:10.1186/s12995-023-00373-7)
Supplement: Supplementary file 5 — Additional file 5. Differences in mean values of HCClog: results from two-tailed t-tests (n=55). [file 12995_2023_373_MOESM5_ESM.pdf]

The association between study conditions and hair cortisol in medical students in Germany – a cross-sectional study

Journal of Occupational Medicine and Toxicology

Meike Heming, Peter Angerer, Jennifer Apolinário-Hagen, Urs Markus Nater, Nadine Skoluda, Jeannette Weber[1]

Corresponding author: Jeannette Weber, Institute of Occupational, Social, and Environmental Medicine, Centre for Health and Society, Faculty of Medicine, Heinrich-Heine University Düsseldorf, Universitätsstr. 1, 40225 Düsseldorf, Germany

Additional file 5. Differences in mean values of HCC_{log}: results from two-tailed t-tests (n=55).

		n	Mean	(SD[a])	t	p-value
**JDCS in university setting[b]**						
Demands	>= median	38	0.74	(.21)	2.84	**.006**
	< median	17	0.56	(.23)		
Decision latitude	>= median	35	0.67	(.21)	-0.85	.400
	< median	20	0.72	(.27)		
Support from students	>= median	32	0.62	(.19)	-2.90	**.005**
	< median	23	0.79	(.25)		
Support from professors/lecturers	>= median	29	0.69	(.22)	0.13	.900
	< median	26	0.68	(.25)		
**Student ERI[c]**						
Effort	>= median	21	0.78	(.23)	2.41	**.020**
	< median	34	0.63	(.22)		
Reward	>= median	20	0.66	(.19)	-0.62	.540
	< median	35	0.70	(.25)		
ER-ratio[d]	>= median	28	0.75	(.24)	2.00	.051
	< median	27	0.63	(.20)		

[a] Standard Deviation.

[b] Structural study conditions questionnaire (In German: StrukStud) (1).

[c] Student version of effort-reward imbalance questionnaire (2).

[d] Effort-Reward Imbalance ratio.

Bold values indicate a p-value below 0.05.

References

1. Schmidt LI, Scheiter F, Neubauer A, Sieverding M. [Demands, Decision Latitude, and Stress Among University Students: Findings on Reliability and Validity of a Questionnaire on Structural Conditions (StrukStud) Based on the Job Content Questionnaire]. Diagnostica 2019; 65(2):63–74.

2. Wege N, Li J, Muth T, Angerer P, Siegrist J. Student ERI: Psychometric properties of a new brief measure of effort-reward imbalance among university students. Journal of psychosomatic research 2017; 94:64–7.
